# Aluminide Diffusion Coatings on IN 718 by Pack Cementation

**DOI:** 10.3390/ma15155453

**Published:** 2022-08-08

**Authors:** Mihai Ovidiu Cojocaru, Mihai Branzei, Leontin Nicolae Druga

**Affiliations:** 1 Department of Metallic Materials Science, Physical Metallurgy, Faculty of Materials Science and Engineering, University POLITEHNICA of Bucharest, 060042 Bucharest, Romania; 2 Section IX-Materials Science and Engineering, Technical Sciences Academy of Romania, 030167 Bucharest, Romania

**Keywords:** aluminide, diffusion coating, pack cementation, Kirkendall–Frenkel effect, pack aluminizing mixture, Al and Ni weight percentage ratio (Al/Ni)

## Abstract

This paper addressed the issues of both direct and indirect synthesis of Ni aluminides by pack cementation (pure Ni and IN 718 superalloy). On the Al-Ni diffusion twosome under pressure, at temperatures below and above the Al melting temperature, the appearance and evolution of diffusion porosity because of the Kirkendall–Frenkel effect manifestation was highlighted. It has been confirmed that, as the temperature rises above the Al melting temperature, the porosity decreases. Nickel-based superalloys, and in particular IN 718, significantly increase their performance by increasing the aluminides proportion in the top diffusion coating. This is made possible by changing the value of the Al and Ni weight percentage ratio in this area (noted as Al/Ni). In the case of the diffusion twosome between IN 718 and pack aluminizing mixtures, having in their composition as active components Al powder, Ferroaluminum (FeAl40) or mixtures of Al and Fe powders, at processing temperatures above the Al melting temperature, by modifying the active component of the mixture, substantial changes in the Al/Ni values were observed, as well as in the maximum %Al in the diffusion coating and of its thickness. It was found that, when switching from Al to FeAl40 or powder mixture (Al + Fe), the Al/Ni value changes between 3.43 and 1.01, from initial subunit values. The experiments confirmed that the highest %Al in the top aluminized diffusion coating, for IN 718, was obtained if the powder mixture contained 66.34 wt.% Al.

## 1. Introduction

Analysis of the Al-Ni phase diagram [1,2,3,4,5], highlights the presence of five nickel aluminides, namely Al_3_Ni; Al_3_Ni_2_; AlNi; Al_3_Ni_5_; and AlNi_3_. In this regard, Okamoto showed, comparing and superimposing in order the binary diagram adopted by Nash et al. (after that of Massalski), in 1991, with that modified by Oka in 1993 (based on the work of Jia in 1991), as well as with that of Ma from 2003, the Al-rich boundary of AlNi_3_ may have to be modified drastically so that it intersect the Ni-rich boundary at its peritectic decomposition temperature. Two of these intermetallic compounds, namely those with a high nickel content (AlNi_3_ and AlNi), are particularly important [6], due to the properties they induce.

Thus, AlNi_3_ acts as a strengthening phase in most superalloys [7], and AlNi, because of its low density, high melting temperature, good thermal conductivity, and oxidation resistance, qualifies as a high-temperature structural material [8].

There is a wide range of applications, such as catalysis, for which, as Hossein, et al. [9] point out, the less studied intermetallic compounds with low Ni concentrations (Al_3_Ni, Al_3_Ni_2_) are of particular importance. In this regard, compositions in the Al-Ni system with high Ni concentrations are of particular interest for use in the field of aviation and space equipment [10,11,12].

Ni-based superalloys of the IN 718 type, with high corrosion resistance, are designed to operate, according to Wakshum, et al. [11], in wide temperature ranges, between minus 250 °C and 704 °C. An increase in the Al percentage in the diffusion coating of the products ensures an increase in the proportion of complex nickel aluminides, especially those of the type AlNi_3_, particularly (Al, Ti)Ni_3_, a phenomenon with direct effects of an increase in the strength characteristics of these areas.

**Figure 1:**
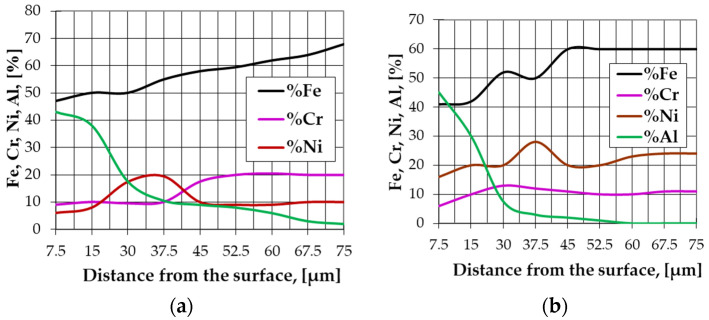
Distribution of Al and alloying elements in the alited layer for an austenitic stainless steel—type 18/8, stabilized with Ti (**a**), and of high refractory alloy steel—0.1% C; 23% Ni; 11% Cr; 3% Ti; 1.3% Mo (**b**).

An increase in the proportion of nickel aluminides in the Ni-base superalloys top diffusion coating of the products can be ensured by their superficial saturation with aluminum, referred to as aluminizing. According to Lahtin and Arzamasov [13], the diffusion of aluminum during the alloying process generates a phenomenon of “pushing” the carbon and the alloying elements from the top diffusion coating to the deeper areas (Figure 1, adapted from Lahtin and Arzamasov 1985 [13]), a phenomenon caused by the low solubility of these elements in aluminides.

Aluminizing is one of the most effective processes to increase the refractoriness, corrosion, and erosion resistance of a wide range of alloys, Ni-based alloys being one of them. Al-supplying media capable of being absorbed in the surface areas of metal products can be solid (powdery or paste), liquid, or gaseous [13,14]. Aluminization by pack cementation is one of the most studied and widespread processes to solve the problem of metal matrices’ surface saturation with Al.

The pack aluminizing mixture usually used has the following three distinct components in terms of their functional role:

1. the active component, suppliers of Al in the active state: frequently aluminum powder (about 50 wt.%), or high-content Ferroaluminum powder (about 40%), which is 98% of the powder mixture;
2. the neutral component, with the role of preserving the uniform distribution of the active component in the entire volume of the powder mixture and at the same time avoiding the tendency of particles’ mutual sintering of the active component during the aluminizing process. Alumina (Al_2_O_3_) is the most frequently used component with the role of dispersing and blocking the sintering tendency. If FeAl40 is used as an active component of the pack aluminizing mixture, it is no longer necessary to use a neutral component alongside the active one;
3. the component with the role of activating the metal surface subjected to processing and, at the same time, directly participating, most often, in the reactions generating aluminum in the active state, is ammonium chloride (NH_4_Cl).

Vilupanur and Chen [15], based on the results of a series of studies [16,17,18], concluded that, depending on the activity, the powdery solid media used for the pack cementation process can be classified into high or low active environments. In the case of high activity media, Al diffuses into the substrate, generating, together with Ni, a solid solution based on an intermetallic compound of the NiAl type. In the case of low activity media, the process of Ni diffusion from the substrate to the outside prevails, a phenomenon that determines a slight increase in the thickness of the parts after aluminization by pack cementation (with 10 ÷ 20 microns, according to Pugacheva [19]).

Nickel-based superalloys are subjected to aluminization by pack cementation to increase working performance. According to Borzdâka and Teitlin [20], the Al/Ni weight percentage ratio and elements that generate hardening phases decisively influence durability and especially plasticity, tested at intervals of thousands of hours. If the alloy also contains Nb, it can be found in carbides or intermetallic phases at higher concentrations, such as the IN 718 superalloy. The IN 718 superalloy falls into the category of alloys in which the Al/Ni weight proportions ratio is subunit. In this case, according to Borzdâka 1964, the products made from it are not recommended for long-term operation.

The metastable phase γ’-Ni_3_(Al, Ti) takes shape in such a superalloy under strictly determined conditions and tends to become more stable over time. At subunit values of the Al/Ti weight proportions ratio, by maintaining the temperature range of 750 ÷ 800 °C, there is a partial separation (elimination) of Ti from the metastable intermetallic compound Ni_3_(Al, Ti), resulting in the formation of independent phases and increasing of the superalloy brittleness [20]. Therefore, alloys with an Al/Ti weight proportion ratio of <<1 are not suitable for long-term operation.

By analyzing the CCT diagrams corresponding to the IN 718 superalloy [21,22], it can be anticipated that, while holding the temperature range at which the aluminizing of Ni-based superalloys frequently occurs, along with the superficial saturation of Al and the appearance in these coating areas of various Ni-aluminides, it can also take shape through the precipitation, in the whole volume, of a γ’’ type phase (metastable phase with a tetragonal crystal structure), δ (stable phase with an orthorhombic crystal structure), or γ’, in correlation with the temperature and the holding time at the aluminizing temperature.

The γ’-Ni_3_(Al, Ti) phase in the IN 718 superalloy has a minor effect on its properties due to its low volume ratio [21], and the γ’’-Ni_3_Nb metastable phase [23,24,25,26,27], at long periods of holding time at temperatures above 700 °C, tends to turn into a stable phase δ-Ni_3_Nb, so that this temperature becomes the upper limit of working temperature of parts made of this superalloy.

Regarding the stable phase δ-Ni_3_Nb, the preferential separation at the grain boundary causes a slowdown of the grain size increase, having positive effects on the properties [21]. Maj [28] showed that the metastable phase γ’’-Ni_3_Nb germinates very homogeneously in the temperature range of 650 ÷ 750 °C (15 ÷ 20% vol.) causing a substantial increase in the resistance of the IN 718 superalloy, and by prolonged exposure to high temperatures, passes in a stable form, δ-Ni_3_Nb.

Changing the Al/Ni weight percentage ratio from subunit to supraunitar, because of the Al surface saturation of the products made of IN 718 superalloy, will involve an increase in Al concentration in the diffusion coating, resulting in a substantial increase in refractoriness due to AlNi type intermetallic compounds [29].

The experimental research in this study was carried out in two directions, as follows:

- Checking the behavior of the Al-Ni diffusion couple (solid or powdery, with Al as an active component) in the conditions of holding temperatures below and above the Al melting temperature;
- Explaining the effects of the phase composition variation of the pack cementation mixture used in the IN 718 superalloy aluminizing, on the level of the Al/Ni weight proportion ratio in the top diffusion coating, the maximum %Al value in the diffusion coating and the diffusion coating thickness.

## 2. Materials and Methods

Of particular interest is the understanding and quantification of the effects of changing the nature of the active component of the pack aluminizing mixture on the Al/Ni weight proportion ratio adsorbed and diffused in the IN 718 superalloy. In this regard, the experimental research considered three types of active components, namely Al powder, and Ferroaluminum (FeAl40) with about 40 wt.% Al, and a mixture of Al and Fe powder in equal proportions by weight. The evolution of the %Al and Al/Ni weight proportion ratio was observed and analyzed in the diffusion coating.

To achieve the objectives imposed by the research topic, a series of raw materials were used, such as metallic, and non-metallic powders, but also compact materials obtained by specific conventional techniques of classical metallurgy (bars, sheets, etc.), as well as techniques for processing and investigating the results obtained.

Achieving the goals of experimental research has become possible by conducting experimental research in two main directions:

- On the diffusion twosome:
  (A_1_)—Massive Al-Ni, under pressure at 100 KPa, at 650 °C, for 50 h—air atmosphere;
  (A_2_)—Massive Ni—powder mixture (Al-Al_2_O_3_ + NH_4_Cl), at 1000 °C for 50 h—air atmosphere;

The evolution of the diffusion porosity generated by the manifestation of the Kirkendall–Frenkel effect and the types of phases that occurred have been studied.

B. On the Ni-based superalloy diffusion twosome/INCONEL 718—powdery solid aluminum supplier, at 900 °C for 5 h;
  (B_1_)—IN 718—powdery solid mixture (Al-Al_2_O_3_ + NH_4_Cl);
  (B_2_)—IN 718—powdery solid mixture (FeAl_40_ + NH_4_Cl);
  (B_3_)—IN 718—powdery solid mixture (Al-Fe + NH_4_Cl).
  Note: the samples were packed in powders mixtures, in refractory steel boxes (300 × 180 × 180 mm) sealed with clay, in a chamber electric furnace with 10 KW, equipped with an automatic system of programming and temperature control.

The evolution of the aluminum gradient concentration, the Al/Ni weight proportion ratio, the phase composition, and the diffusion coating thickness have been studied.

The powdery materials for pack cementation used in the experimental research were the following:

- Al powder, produced by air spraying in Zlatna (Romania), by the Sherritt hydrometallurgical process, of 99.2% purity (0.15% Fe, 15% O_2_; 0.5% N_2_) with an average particle diameter of about 50 μm;
- Fe powder, produced in Höganäs (Sweden), obtained by atomization, with a purity greater than 99.9% and an average diameter of about 45 μm;
- Ferroaluminum (FeAl40) produced by Avon Metals LTD (UK), fragments with an average equivalent diameter of 40 ÷ 50 mm, subsequently ground in ball mills up to equivalent average diameters of 3 ÷ 4 mm;
- Alumina powders, with a purity greater than 98.5%, produced by Alum Tulcea S.A. (Romania), having a maximum 10% for the fraction greater than 150 μm and 12% for the fraction less than 45 μm;
- Ammonium chloride powder of analytical purity, produced by Silver Chemicals (Romania);
- The obtained results involved the following investigations:
- Determination of the oxygen and nitrogen content of the powders used, by ASTM E 1019-11, using the LECO TC-236;
- Hardness testing using a Rockwell Insize ISH-R150 manual hardness tester;
- Highlighting microstructural changes by optical microscopy (Image Analysis System—Buehler Omnimet), scanning electron microscopy (TESCAN VEGA XMU 8 and Philips XL30 ESEM TMP), and EDAX analysis (EDAX Sapphire type dispersive energy spectrometer with the resolution of 128 kV), as well as X-ray diffraction (POWDER X-RAY DIFFRACTOMETER—GNR APD 2000 PRO and D8 Advanced type—BRUKER-AXS).

## 3. Results and Discussion

(A-A_1_): The profile technical literature shows significant differences between the values of the diffusion coefficients of aluminum in nickel [30,31], and vice versa [32,33,34], as shown in Table 1, being about eight orders of magnitude if the phenomenon occurs below the Al melting temperature, and six orders for temperatures above this value.

**Figure 2:**
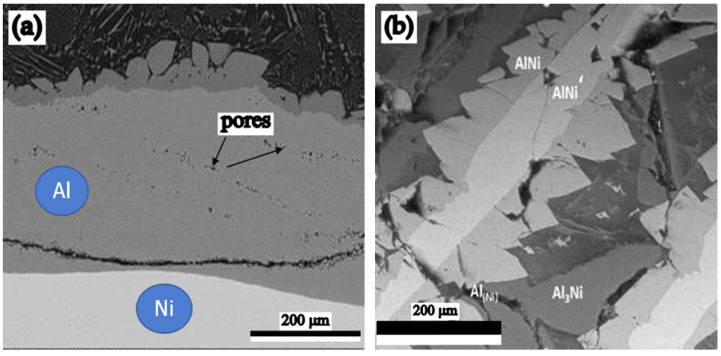
The diffusion porosity generated by the Kirkendall–Frenkel effect in the Al-Ni diffusion couple after holding for 50 h at 650 °C (**a**) [35], and the presence of Al_3_Ni aluminide highlighted in the SEM image performed in the Al matrix after holding at 1000 °C for 20 h, in contact with the Ni matrix (**b**) [36].

**Figure 3:**
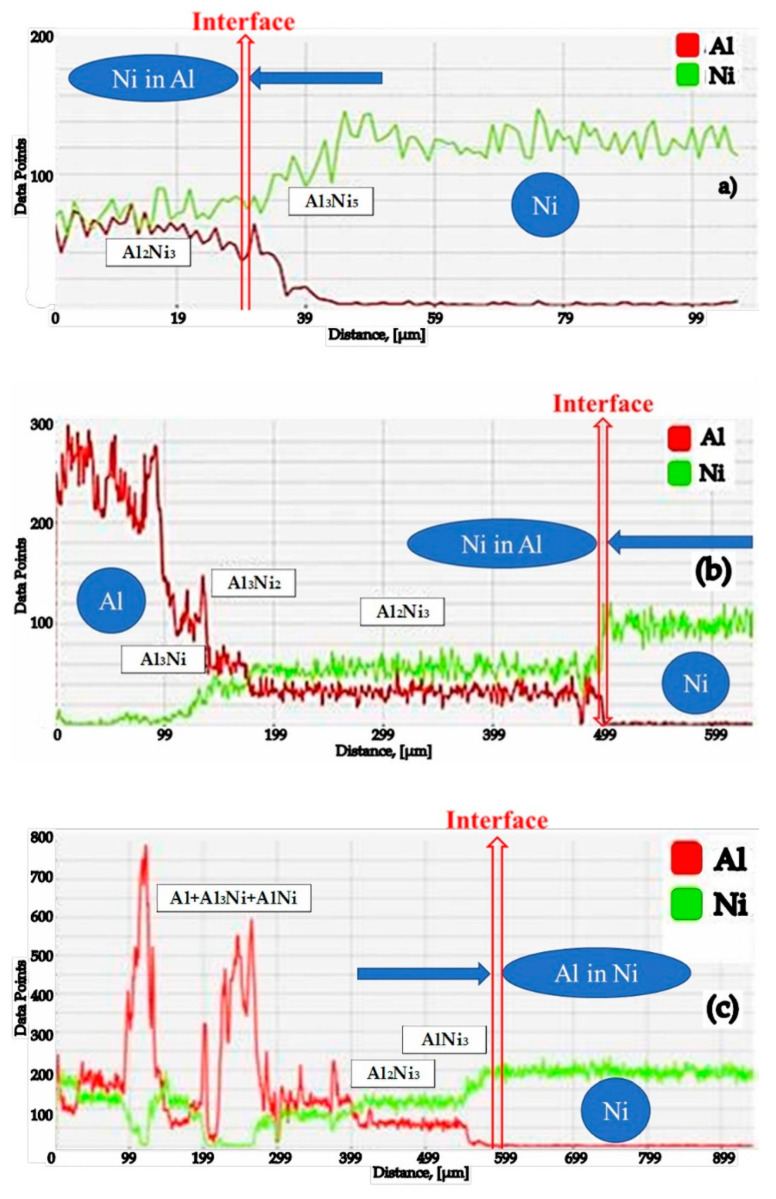
The effects of Ni cementation in the pack cementation process (50 wt.% Al + 49 wt.% Al_2_O_3_ + 1 wt.% NH_4_Cl), at 650 °C for 20 h (**a**) and at 1000 °C for 20 h (**c**); Al and Ni distribution in the Al-Ni diffusion couple at 100 KPa and at 650 °C for 50 h (**b**).

Therefore, the Kirkendall–Frenkel effect was manifested in the case of these diffusion couples, as expressed by the appearance of diffusion porosity (Figure 2a) [35].

(A_2_): With the increase of the temperature at which aluminizing occurs above the Al melting temperature, the diffusion couple consists of bulk Ni and powdery Al representing the active component (50% of the solid powdered alloy mixture). This is affected by a decrease in the level of diffusion porosity, due to the tendency of the micropores to fill, by capillarity, with Al molten and degradation of the Ni matrix [36] on contact with molten aluminum (Figure 3c), a phenomenon caused by the Al_3_Ni aluminide synthesis, whose melting temperature is below that of the microzones in which it is formed (854 °C, compared to 1000 °C).

The actual temperature of the microzones in which the Al_3_Ni compound appears is much higher than its melting temperature, due to the substantial caloric contribution generated by its fade in (ΔH_1000°C_ = −191.7 kJ/mol) [37]. Over time, the Ni-Al front moves in the nickel matrix (degradation advances).

Between the two elements of the diffusion couple, Al (from the solid powder A_2_, or bulk mixture A_1_) and Ni, during the aluminides synthesis processes, due to the interdiffusion phenomena and regardless of temperature, a compositional gradient appears, which results in a continuous variation of the aluminides type (Figure 3), the pack cementation process (50 wt.% Al + 49 wt.% Al_2_O_3_ + 1 wt.% NH_4_Cl), at 650 °C for 20 h (a) and at 1000 °C for 20 h (c); Al and Ni distribution in the Al-Ni diffusion couple at 100 KPa and at 650 °C for 50 h (b).

In the Al-Ni diffusion twosome, the two elements of the couple under pressure at 100 KPa, at a temperature below the Al melting temperature (660 °C, for 50 h), were identified as a series of phases, as follows: Al_3_Ni; Al_3_Ni_2_; Al_2_Ni_3_—phase with reduced thermodynamic stability (see Figure 3b), determined by the predominant diffusion of Ni in Al. This is explained by the existence of differences of about eight orders of magnitude between the reciprocal diffusion coefficients of the two elements.

**Figure 4:**
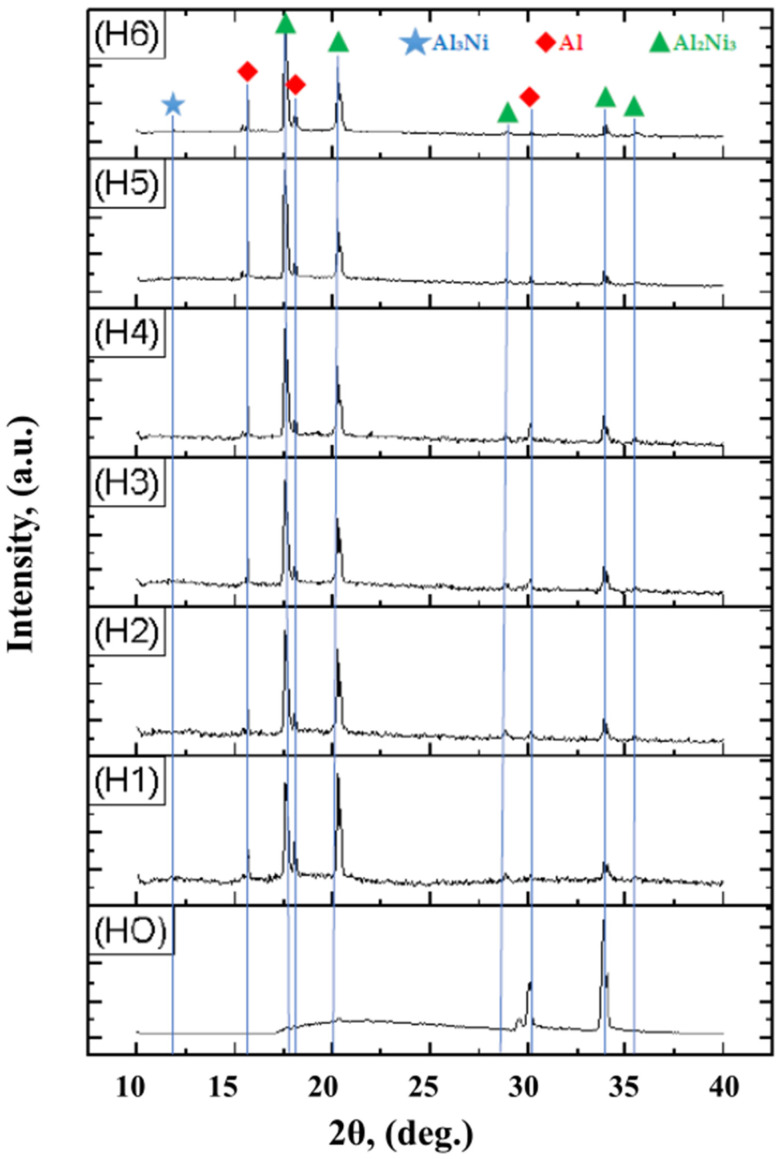
X-ray diffraction patterns taken at different depths (H1 ÷ H6) in relation to the surface of the Al sample (H0 = 0 mm), in the case of an Al-Ni diffusion couple, holding at 650 °C for 50 h; (HO) = 0/surface; (H1) = 0.65 mm; (H2) = 1.15 mm; (H3) = 1.27 mm; (H4) = 1.37 mm; (H5) = 1.55 mm; (H6) = 1.75 mm.

As shown in Figure 4, X-ray diffraction of the Al-Ni diffusion couple in the Al area identified the Al_2_Ni_3_ phase, a pentanucleic, intermetallic cluster with a composition that can serve as a precursor for the formation of nanoparticles. The thermodynamic stability of this metallic pentanucleic cluster is reduced so that it will decompose relatively easily after the disproportionate reaction: Al_2_Ni_3_ = Al + AlNi_3_.

**Figure 5:**
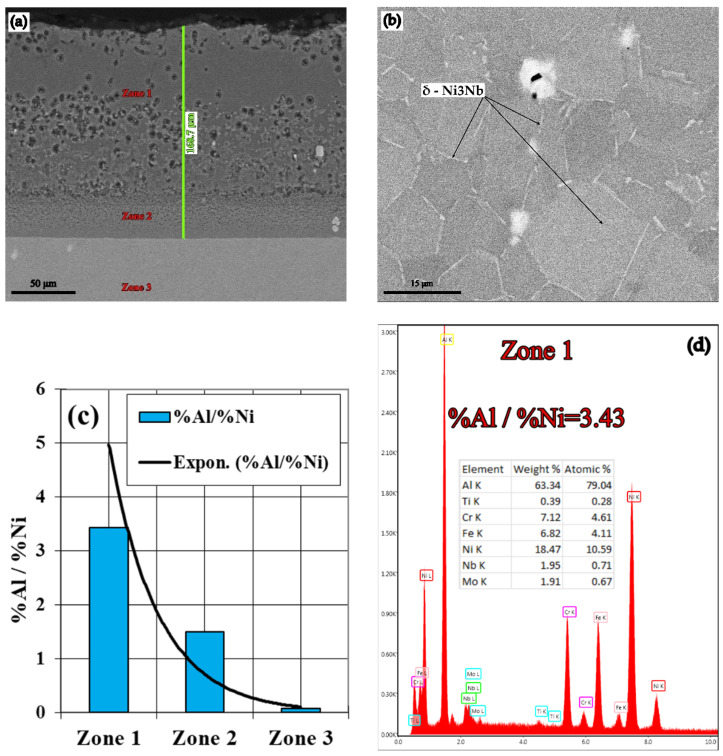

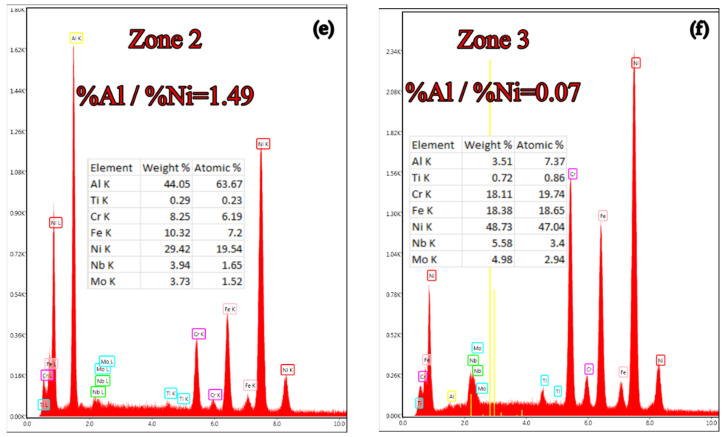
Aluminized IN 718 superalloy (900 °C/5 h), in a pack aluminizing mixture (50 wt.% Al + 49 wt.% Al_2_O_3_ +1wt.% NH_4_Cl); SEM-EDS spectra corresponding to the different areas investigated, and the %Al/%Ni ratio in the investigated areas.

**Figure 6:**
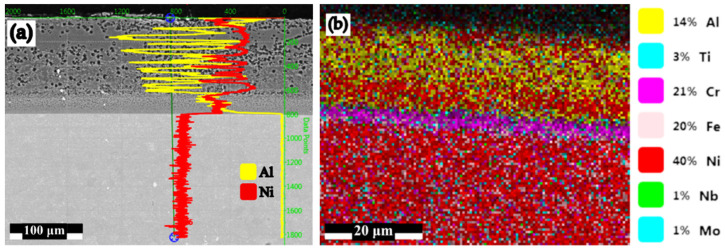
Al and Ni profile plot for a pack aluminized IN 718 superalloy (900 °C/5 h) in pack aluminizing mixture (50 wt.% Al + 49 wt.% Al_2_O_3_ + 1 wt.% NH_4_Cl) (**a**); the main alloying elements distribution (**b**).

The appearance of intermetallic compounds causes a substantial change in the values of the Al diffusion coefficient in Ni-based metal matrices. Thus, according to Cserhati et al. [38], in the presence of the intermetallic compound Ni_3_Al, the diffusion coefficient of aluminum decreases to 650 °C, with an order of magnitude in relation to the one calculated at this temperature for its diffusion in the Ni matrix (reaches 8.8 × 10^−21^ m^2^/s, compared to 6.25 × 10^−20^ m^2^/s), and with two orders of magnitude at 1000 °C (reaches 5.3 × 10^−17^ m^2^/s, compared to 1.37 × 10^−15^ m^2^/s).

(B-B_1_): The IN 718 superalloy pack aluminizing at 900 °C for 5 h (Figure 5 and Figure 6), in a pack aluminizing mixture containing 50 wt.% Al powder, 49 wt.% Al_2_O_3_ powder, (as a protective factor against mutual sintering of Al particles), and 1 wt.% NH_4_Cl, (as an activator of the surfaces and at the same time as the reactions between the components of the environment), determined an increase of the Al concentration in the top diffusion coating, up to values of 63.34 wt.%, thus bringing the value of the Al/Ni weight proportion ratio in these zones, to 3.43 (Zone 1, Figure 5a). The stable phase δ-Ni_3_Nb (see Figure 5b), is separated at the grain boundaries, as Chandler showed in his paper [21].

The permanently decreasing variation of the Al content in the diffusion coating is reflected both in the mode of the Al/Ni ratio variation (Figure 5c,d, 1.49 in Zone 2 (44.05 wt.% Al) and, 0.07 in Zone 3 (3.51 wt.% Al), as is shown in Figure 5e,f). The distribution profile of the concentrations of the two elements in the diffusion coating is presented in Figure 6a. The distribution of the main alloying elements in IN 718 diffusion coating and substrate is presented in Figure 6b.

**Figure 7:**
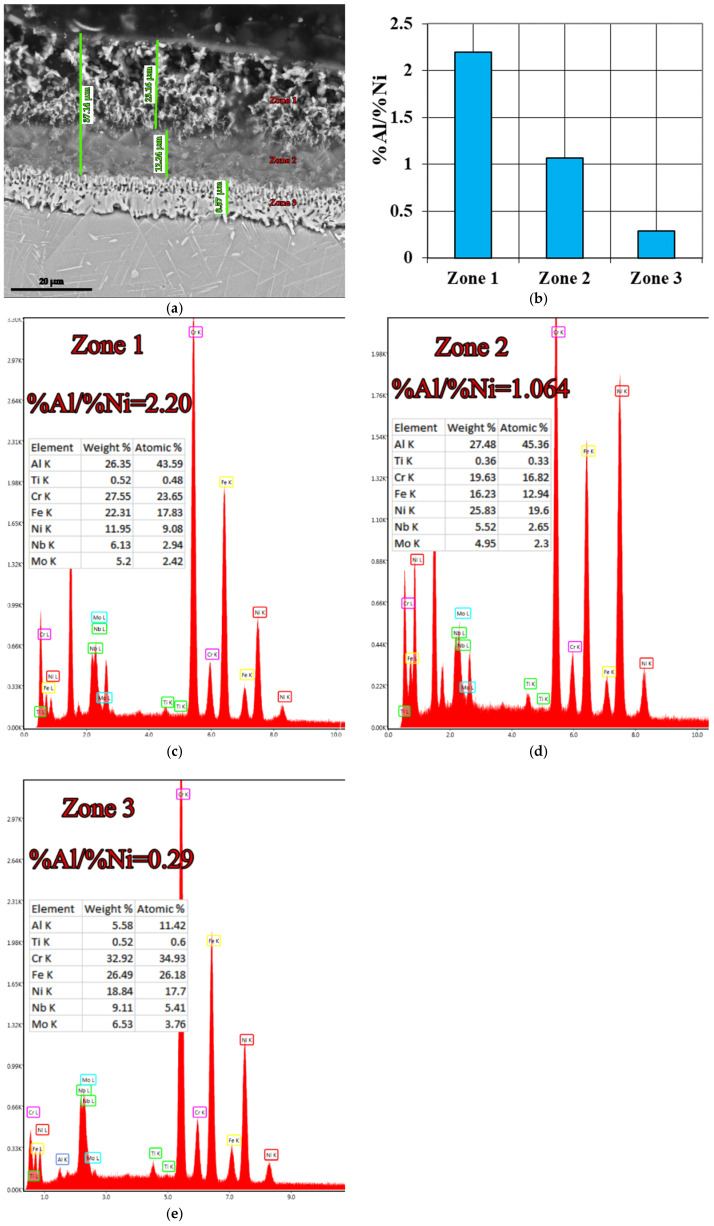
Aluminized Inconel 718 superalloy at 900 °C for 5 h, in a pack aluminizing mixture (98 wt.% FeAl40 + 2 wt.% NH_4_Cl) (**a**); %Al/%Ni weight proportion ratio variation in the investigated zones (**b**); SEM-EDS spectra corresponding to the different zones investigated (**c**–**e**).

**Figure 8:**
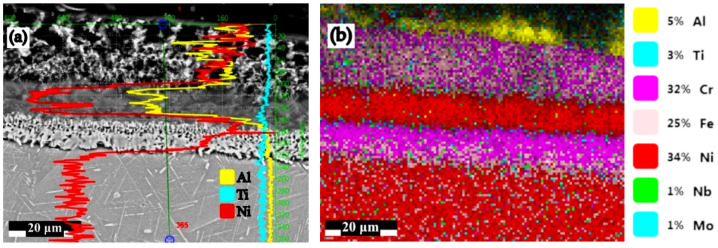
Al and Ni profile plot for pack aluminized IN 718 superalloy (900 °C/5 h) in pack aluminizing mixture (98 wt.% FeAl40 + 2 wt.% NH_4_Cl) (**a**); the main alloying elements distribution (**b**).

The high activity of the pack aluminizing mixture used for the Ni-based superalloy aluminizing caused the diffusion porosity to be located mainly in the diffusion coating, in the areas rich in Al where Al_3_Ni and AlNi type intermetallic compounds are also found. The high concentration of Al in these areas also induces a high brittleness of the coating, which leads to the need to use the powdery composition instead of Al powder or Ferroaluminum powder with moderate %Al content (about 40 wt.% Al)—FeAl40.

(B_2_): The IN 718 superalloy pack cementation in media containing 98 wt.% FeAl40 and 2 wt.% NH_4_Cl (Figure 7 and Figure 8) resulted in a change in the Al/Ni weight proportion ratio, from subunit to supraunitary, i.e., 2.20, given that the %Al in the top area of the diffusion coating reaches a maximum of about 27.5%, a value at which, theoretically, there is no embrittlement of the coating. Therefore, no further technological measures are needed after the aluminizing to reduce the level of concentration of this element (for example, subsequent annealing).

Regardless of the type of active component Al supplying the cross-section of the diffusion coating, the concentration continuously decreases; at the same time, the effect of “pushing” the alloying elements (Figure 1) [13] to deeper areas was noticed, which was more accentuated in the case of very active media (those in which the active component is Al powder) and less accentuated in the other studied cases.

It should be noted that holding the temperature at 900 °C, for 5 h, followed by slow cooling, favors the precipitation of the hardening phase δ-Ni_3_Nb (Figure 5b), inside the grains of solid solution γ, a phenomenon that ensures an increase in macro hardness on average by about 15 Rockwell units (from about 25HRC to about 40HRC after pack cementation).

(B_3_): In the experimental research, the mixture of Al and Fe powder in equal proportions by weight was used as an alternative to the active component of the pack aluminizing mixture. We assumed that thermodynamically, during heating and holding the cementation temperature, the components of the powdery solid mixture are very probably chemical interactions with the formation of iron aluminides, existing in Ferroaluminum, as shown Begunov et al. [39].

**Figure 9:**
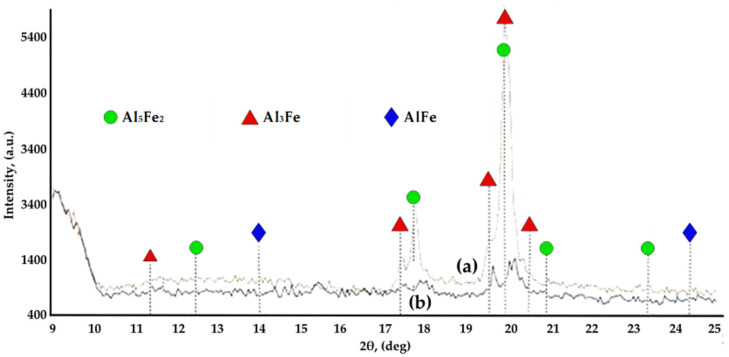
X-ray diffraction analysis, after the pack cementation process (900 °C/5 h), of the specimens from the two compositions used as active components: (**a**) FeAl40 powder; (**b**) the mixture of Al and Fe powder in equal proportions by weight.

X-ray diffraction analysis (Figure 9) of the specimens from the two compositions used as active components of the pack cementation process, FeAl40 and the mixture of Al and Fe powder in equal proportions by weight, highlighted the presence of Fe aluminides AlFe, Al_5_Fe_2,_ and Al_3_Fe, the main suppliers of Al during the alloying process, in both types of mixtures.

**Figure 10:**
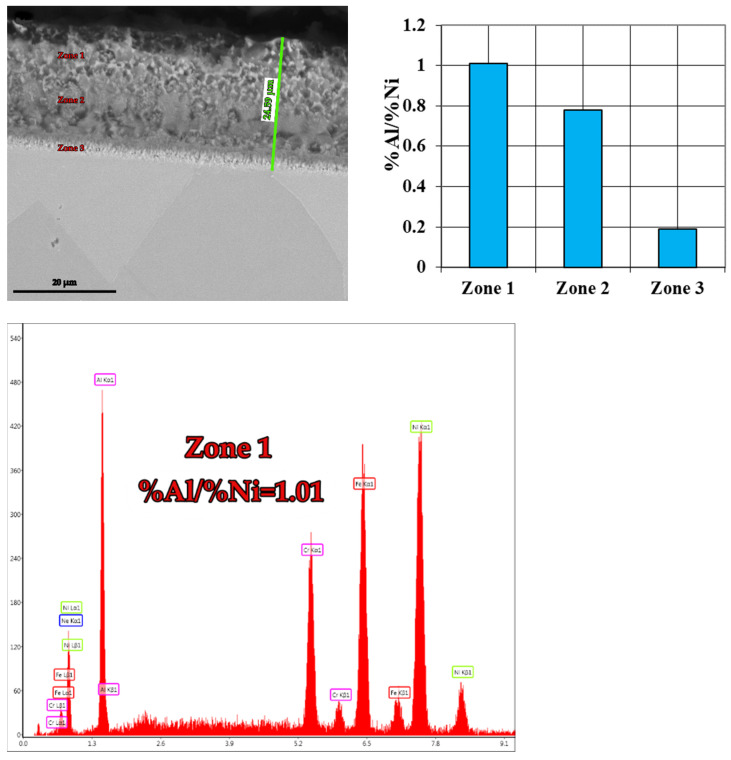

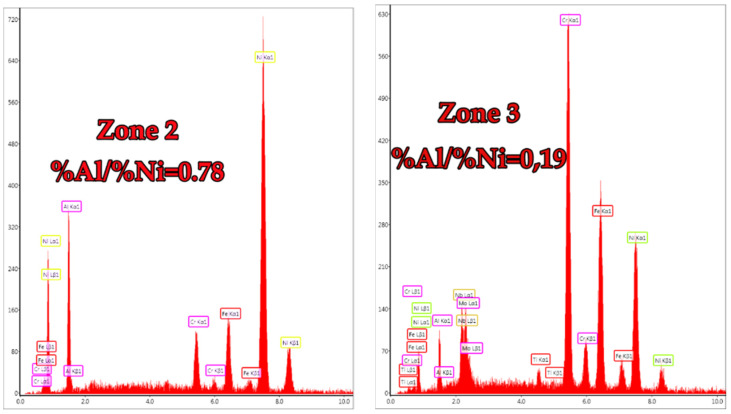
Aluminized IN 718 superalloy at 900 °C for 5 h, in a pack aluminizing mixture (49 wt.% Al + 49 wt.% Fe + 2 wt.% NH_4_Cl); SEM-EDS spectra corresponding to the different areas investigated, and the %Al/%Ni ratio in the investigated areas.

**Figure 11:**
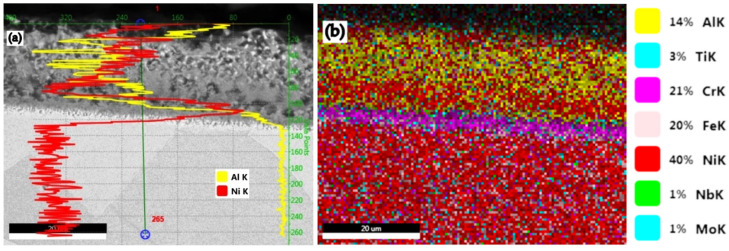
Al and Ni profile plot for pack aluminized IN 718 superalloy (900 °C/5 h) in a pack aluminizing mixture (49 wt.% Al + 49 wt.% Fe + 2 wt.% NH_4_Cl) (**a**); the main alloying elements distribution (**b**).

Experimental research carried out with the mixture of Al and Fe powder in equal proportions by weight as a pack cementation mixture confirmed the theoretical hypotheses (see Figure 10 and Figure 11), that, in the top diffusion coating, the %Al reached about 36.61%, and values of the Al/Ni ratio of about 1.01 (compared to 2.20 in the case of FeAl40 use).

**Figure 12:**
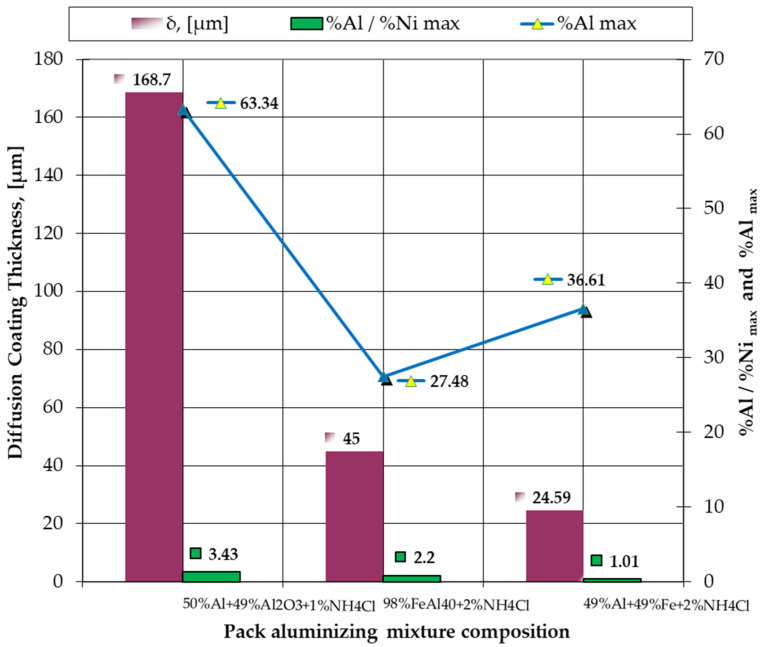
The effects of the change in the phase composition of the pack aluminizing mixture on the diffusion coating thickness and the %Al/%Ni ratio in the top diffusion coating on IN 718 by pack cementation; pack cementation process: holding at 900 °C for 5 h, and slow cooling.

Changing the activity of the pack aluminizing mixture also changes the diffusion coating thickness (Table 2 and Figure 12). Thus, it is found that, with the decrease of the activity of the pack aluminizing mixture, there is a strong decrease in the coating thickness; the greatest coating thickness is obtained in the case of the most active mixture (about 169 μm if the pack aluminizing mixture contains 50% free Al), and the transition to FeAl40 or the mixture of Al and Fe powder in equal proportions by weight generates a decrease in the diffusion coating thickness to an average value of about 45 μm and 25 μm, respectively.

It should be noted that this pack cementation process, with slow cooling, in the pack aluminizing mixture, favors precipitation of the δ-Ni_3_Nb hardening phase (Figure 5b) inside the grains of solid solution γ, a phenomenon that increases the matrix hardness by an average of about 15 HRC units (from 25HRC initially to about 40 HRC).

A comparative analysis of the results of pack cementation with the active components in various states (free, bonded or in the bonded process) of the IN 718 superalloy—series B—is presented in Table 2 and in more detail in Figure 12.

The decrease in the value of the Al/Ni weight proportion ratio is related to the decrease in the activity of the pack aluminizing mixture, reflected in the maximum concentration of Al in the diffusion coating, and has an immediate effect on the diffusion coating thickness.

## 4. Conclusions

The conclusions are grouped according to the two research directions mentioned at the end of the introductory section, as follows:

- The differences between the values of the diffusion coefficients of Al in Ni, and vice versa, cause the Kirkendall–Frenkel effect to be manifested in this diffusion couple. Increasing the diffusion couple temperature has the effect of reducing the porosity.
- Between the two elements of the diffusion couple, during the process of aluminide synthesis and regardless of the temperature, a compositional gradient appears, which has as a consequence a continuous variation of the aluminide types.
- All the powder mixtures compositions allowed, by aluminizing and by pack cementation, some supraunitary values of the %Al/%Ni weight proportion ratio to the top diffusion coating of the IN 718 superalloy to be achieved.
  (1) The activity of the pack aluminizing mixture used is strictly dependent on the state in which the Al is found in the environment (free or bonded).
  (2) The most notable effects are obtained when pack cementing with free aluminum, alumina, and ammonium chloride. Thus, at 900 °C, with a 5 h holding time, a higher diffusion coating thickness is generated (168.7 microns), with the concentration of aluminum in the layer also being at the highest level (63.34 %Al). In this case, the %Al/%Ni weight proportion ratio is 3.43.
  (3) The layers obtained after pack aluminizing, on an Inconel 718 superalloy matrix, and regardless of the variation in the composition of the pack aluminizing mixture used, are perfectly adherent because they are formed “in situ”.
  (4) The Rockwell macro hardness tests revealed an increase of approximately 15 units (from 25 HRC to 40 HRC) in the case of 900 °C holding time heat treatment temperature, above the melting temperature of Al, for 5 h. This increase is due to the massive precipitation of the δ—Ni_3_Nb hardening phase, in the γ solid solution grains (see Figure 5b).
  (5) The increase in the working performances of the parts made of IN 718 superalloy grade can be ensured by their pack cementation. The composition of the powdery mixture consisting of an aluminum powder dispersed in an alumina powder is recommended, as this leads to the formation of the maximum layer thickness, as well as the highest value of the %Al/%Ni weight proportion ratio in the area adjacent to the surface (for the same processing conditions); under these conditions, high resistance and refractoriness of the layer are obtained, for long-term demands. This variant of thermochemical processing does not require specialized equipment or operating personnel with special skills. Heating equipment and refractory steel boxes are required for packing parts into the pack aluminizing mixture.
- The described technological process variants are environmentally friendly, both in terms of the materials used and the resulting reaction products.

## Figures and Tables

**Table 1:** Values of diffusion coefficients of Al in Ni andNi in Al.

Under Al Meting Temp.—650 °C		Over Al Melting Temp.—1000 °C	
Ni in Al diffusion [m^2^/s]	2.65 × 10^−12^ [32]	Ni in Al diffusion [m^2^/s]	2.86 × 10^−9^ [33] & 8.18 × 10^−9^ [34]
Al in Ni diffusion [m^2^/s]	6.25 × 10^−20^ [30]	Al in Ni diffusion [m^2^/s]	1.37 × 10^−15^ [31]

**Table 2:** Comparative analysis of the results of pack cementation with the active components in various states (free, bonded or in the bonded process) of the IN 718 superalloy (processing parameters: 900 °C/5 h).

Pack Aluminizing Mixture Composition	Al_max_ in the Coating, [%]	%Al/%Ni	δ_max_, [μm]
B_1_ → 50%Al + 49%Al_2_O_3_ + 1%NH_4_Cl	63.34	3.43	168.70
B_2_ → 8%FeAl40 + 2%NH_4_Cl	27.48	2.20	45.00
B_3_ → 49%Al + 49%Fe + 2%NH_4_Cl	36.61	1.01	24.59

## Data Availability

The study did not report any data.
